# Flame‐Resistant Inorganic Films by Self‐Assembly of Clay Nanotubes and their Conversion to Geopolymer for CO_2_ Capture

**DOI:** 10.1002/smll.202406812

**Published:** 2024-10-07

**Authors:** Alessandro Lo Bianco, Martina Maria Calvino, Giuseppe Cavallaro, Lorenzo Lisuzzo, Pooria Pasbakhsh, Stefana Milioto, Giuseppe Lazzara, Yuri Lvov

**Affiliations:** ^1^ Department of Physics and Chemistry – Emilio Segrè University of Palermo Viale delle Scienze 17 Palermo 90128 Italy; ^2^ Department of Infrastructure Engineering Faculty of Engineering and Information Technology The University of Melbourne Melbourne Victoria 3010 Australia; ^3^ Department of Mechanical Engineering School of Engineering Monash University Sunway Campus Bandar Sunway Subang Jaya Selangor 47500 Malaysia; ^4^ Institute for Micromanufacturing Louisiana Tech University 505 Tech Drive Ruston LA 71272 USA

**Keywords:** ceramics, CO_2_ capture, geopolymers, halloysite, inorganic films

## Abstract

Self‐assembling of very long natural clay nanotubes represents a powerful strategy to fabricate thermo‐stable inorganic thin films suitable for environmental applications. In this work, self‐standing films with variable thicknesses (from 60 to 300 µm) are prepared by the entanglement of 20–30 µm length Patch halloysite clay nanotubes (PT_Hal), which interconnect into fibrosus structures. The thickness of the films is crucial to confer specific properties like transparency, mechanical resistance, and water uptake. Despite its completely inorganic composition, the thickest nanoclay film possesses elasticity comparable with polymeric materials as evidenced by its Young's modulus (ca. 1710 MPa). All PT_Hal‐based films are fire resistant and stable under high temperature conditions preventing flame propagation. After their direct flame exposure, produced films do not show neither deterioration effects nor macroscopic alterations. PT_Hal films are employed as precursors for the development of functional materials by alkaline activation and thermal treatment, which generate highly porous geopolymers or ceramics with a compact morphology. Due to its high porosity, geopolymer can be promising for CO_2_ capture. As compared to the corresponding inorganic film, the CO_2_ adsorption efficiency is doubled for the halloysite geopolymeric materials highlighting their potential use as a sorbent.

## Graphical abstract

Free‐standing inorganic films with variable thicknesses (from 60 to 300 µm) are fabricated by interconnection and tangling of long halloysite clay nanotubes using the aqueous casting procedure. The fabricated films are fire‐resistant and elastic. Thermal treatment and alkaline activation of halloysite films are employed to prepare ceramics and high porous geopolymers efficient for CO_2_ capture.

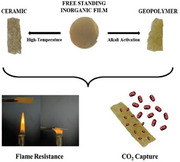

## 1 Introduction

The development of smart materials with tailored properties for environmental purposes is a challenging task for scientific and technological research. Halloysite clay nanotubes are suitable for various biotechnological,^[^ ^1^, ^2^, ^3^, ^4^, ^5^, ^6^ ^]^ environmental,^[^ ^7^, ^8^, ^9^, ^10^ ^]^ catalysis^[^ ^11^, ^12^, ^13^, ^14^ ^]^ and industrial applications.^[^ ^15^, ^16^, ^17^, ^18^, ^19^, ^20^ ^]^ Halloysite (Hal) is a natural nanoclay with a distinctive hollow tubular structure and different surface chemistry that results in an inner positively charged lumen and an external negatively charged surface^[^ ^21^ ^]^ enabling selective loading and release of active molecules.^[^ ^22^, ^23^, ^24^, ^25^ ^]^ Commercially available halloysite nanotubes have diameters from 20 to 200 nm, internal diameters from 10 to 50 nm, and a length ≈1 µm.^[^ ^26^ ^]^ Among clay nanotubes, Patch halloysite (PT_Hal), sourced in Western Australia, presents high purity and is much longer making their performance similar to fibers.^[^ ^27^ ^]^ Their length can achieve up to 30 µm, which makes the length to diameter ratio of ca 200: 1. Such PT_Hal can form a tangled fibrous network enhancing the mechanical and thermal properties of the resulting composite materials^[^ ^28^ ^]^ and leading to the formation of hydrogel even at low concentration.^[^ ^29^ ^]^ The Patch halloysite capacity to form a bird nest structure might be promising for the formation of a completely inorganic composition locked by the interconnection of these long nanotubes.

Halloysite nanotubes are generally safe for biomedical applications, such as oral drug delivery and tissue scaffolds, as they only show toxicity at concentrations over 1000 µg mL^−1^.^[^ ^30^ ^]^ The toxicity level is related to the origin of halloysite. Literature reports that Patch halloysite possesses lower toxicity as compared to halloysites from other geological deposits.^[^ ^31^ ^]^

An interest to completely inorganic films has grown due to their superior mechanical strength, chemical resistance, and thermal stability as compared to organic‐based films.^[^ ^32^ ^]^ They can be exploited in a wide range of applications, including the production of thermoelectric devices^[^ ^33^ ^]^ and solar cells.^[^ ^34^ ^]^ However, traditional technology gives fragile and very thin inorganic films (ca. 1 µm).^[^ ^35^ ^]^ A common chemical vapor deposition^[^ ^36^ ^]^ is more complex than classic solvent casting used for polymeric films^[^ ^37^ ^]^ since they require precise control of environmental conditions and high temperatures, making them less accessible and more expensive.

In addition to use in inorganic films bound by these long clay fibers interaction, Patch halloysite nanotubes can be starting materials for the fabrication of alumino‐silicate geopolymers. Geopolymers are inorganic polymeric materials with Al to Si ratio of 1:1 which is close to halloysite composition, that exhibit excellent mechanical properties, such as compressive strength, very high flame retardancy, and insulating properties,^[^ ^38^ ^]^ making them good potential substitutes for Portland concrete. Geopolymers offer significant environmental benefits, as their production generates significantly lower CO_2_ emissions as compared to traditional cement that is responsible for the release of 5–7% of global CO_2_ emissions^[^ ^39^, ^40^ ^]^ Additionally they can incorporate industrial byproducts such as fly ash and slag, further enhancing their environmental efficiency.^[^ ^41^ ^]^ Geopolymers are formed through reaction of a proper choice of alumina and silicate materials with a strong alkaline solution, resulting in a 3D network of silico‐aluminate structures.^[^ ^42^ ^]^ Historically, ceramic materials have always played a crucial role in human life^[^ ^43^ ^]^ and in recent times they have applications in many fields such as a special strength construction industry, aerospace launching spots,^[^ ^44^ ^]^ biomedical implant engineering,^[^ ^45^ ^]^ and electronics.^[^ ^46^, ^47^ ^]^ Typically, ceramics are produced by exposing clay materials to high temperatures, which induce physical and chemical changes that enhance their mechanical and thermal properties.^[^ ^48^ ^]^

**Figure 1:**
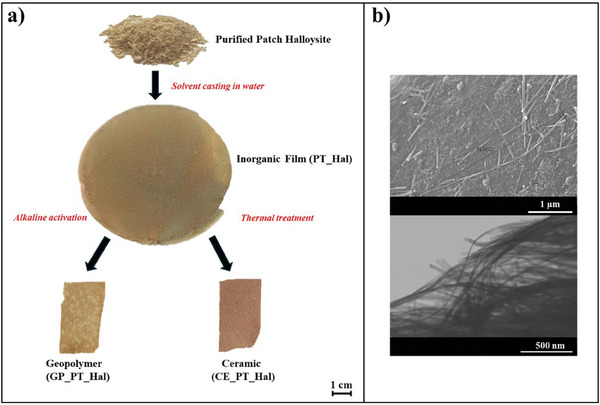
Fabrication of inorganic films, geopolymers, and ceramics based on long Patch halloysite nanotubes. a) Schematic illustration of the preparation of inorganic film based on Patch halloysite (PT_Hal) and subsequent conversion to geopolymer (GP_PT_Hal) and ceramic (CE_PT_Hal). b) SEM and STEM micrographs of purified Patch halloysite nanotubes.

Here, novel inorganic films were developed by solvent casting procedure of purified superlong Patch Halloysite nanotubes (PT‐Hal) as sketched in **Figure** **1**. PT‐Hal films were further employed as precursors to obtain ceramics (CE_PT_Hal) and porous geopolymers (GP_PT_Hal) with high CO_2_ storage capacity. As illustrated in Figure 1a, the conversion of PT‐Hal to geopolymer GP_PT_Hal was achieved by alkaline activation of halloysite, while ceramic CE_PT_Hal was fabricated by high‐temperature treatment of the precursor nanoclay films. Details on the halloysite purification, PT‐Hal film preparation, and subsequent treatments for the fabrication of geopolymer and ceramics are reported in the Experimental Section.

## 2 Results and Discussion

### 2.1 Inorganic Films Based on Patch Halloysite Nanotubes

#### 2.1.1 Morphology and Hydrophilicity

**Figure 2:**
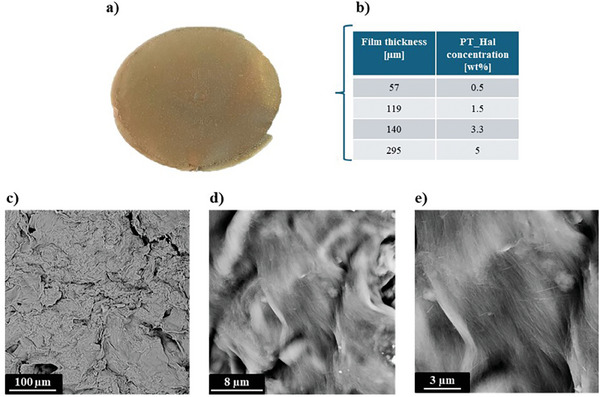
Morphological characteristics of inorganic film based on Patch halloysite nanotubes. a) Optical photo of PT_Hal film with the largest thickness (295 µm). b) Correlation between the film thickness and the PT_Hal concentration of the aqueous dispersions used for the casting protocol. c–e) SEM images of PT_Hal based film with the largest thickness (295 µm) at different magnifications showing the fibrous arrangement of patch halloysite nanotubes within the inorganic film.

**Figure** **2**a shows the optical photograph of PT_Hal based film of 4.5 cm diameter obtained by the solvent casting from 5 wt.% aqueous dispersion. We can control the thickness of the films by changing the PT_Hal concentration in dispersions, and thicknesses ranged from 57 to 295 µm. Such inorganic films appeared to be compact and mechanically strong. These characteristics can be correlated to the microscopic structure of tangled clay nanofibers, as imaged by Scanning Electron Microscopy (SEM) (Figure 2c–e).

It was observed that the film surface is irregular with the presence of halloysite nanotubes arranged into fibrous bundles that extend for tens of microns. Similarly to PT_Hal hydrogels and pectin/PT_Hal nanocomposites,^[^ ^28^, ^49^ ^]^ SEM micrographs show that the inorganic film has a bird nest structure with high entanglement between halloysite nanotubes.^[^ ^28^, ^49^ ^]^ This contributes to the formation of the compact inorganic film, which was not obtained by replacing PT_Hal with shorter 1 µm length halloysite nanotubes like extracted from Dragon Mine source (Figure S1, Supporting Information). The packing effect observed for Patch halloysite nanotubes is related to their length (up to 30 µm), as well as their small thickness. It is noteworthy that composite films based on polymers and shorter halloysite nanotubes (commercially available from Dragon Mine, or Matauri Bay) do not possess fibrous structures with closely packed bundles.^[^ ^50^, ^51^ ^]^

**Figure 3:**
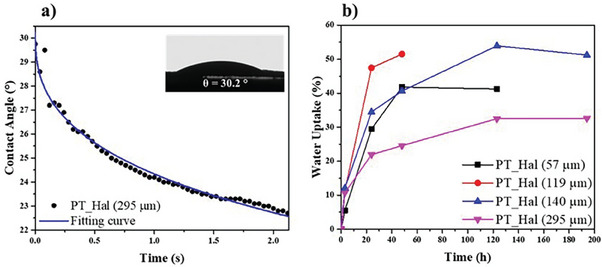
Interactions of PT_Hal film with water molecules. a) Water contact angle as a function of time for PT_Hal film prepared by the 5 wt.% aqueous dispersion. The blue curve is the fitting according to Equation (1). b) Water uptake as a function of time for PT_Hal films prepared by casting dispersions with variable halloysite concentrations (0.5, 1.5, 3.3, and 5 wt.%).

As highlighted by the wettability experiments (**Figure** **3**a), the PT_Hal film has a hydrophilic surface with an initial water contact angle of 30.2°, which is similar to that reported for commercial halloysite compressed tablets (30.7°).^[^ ^52^ ^]^ The high surface hydrophilicity reflects the halloysite chemical composition (Al_2_(OH)_4_Si_2_O_5_·nH_2_O), which makes clay nanotubes suitable for the fabrication of materials with flame resistance capacity.^[^ ^53^, ^54^, ^55^ ^]^ It should be noted that other aluminosilicate clay minerals, such as kaolinite^[^ ^56^ ^]^ and montmorillonite,^[^ ^57^ ^]^ are effective in the production of anti‐flame materials.

The time dependence of the water contact angle (θ) showed a mono‐exponential decay function (Figure 3a) similar to thin films of polymer/nanoclay hybrids.^[^ ^58^, ^59^ ^]^

The kinetic evolution of the water contact angle on PT_Hal film was fitted by using Equation (1):

(1) $$\theta =\theta_{i}\;\exp\left(-k_{\theta }\tau^{n}\right)$$

where *θ*_i_ represents the initial contact angle at *t* = 0, *k*_θ_ is a constant associated with the process rate and *n* is a coefficient ranging between 0 and 1 in dependence of specific absorption/spreading contributions.

According to the PT‐Hal hydrophilicity, the calculated *n* value (0.39 ± 0.02) evidenced that the interactions between the film surface and the water droplet are mostly driven by absorption process, while the *k*_θ_ value (0.216 ± 0.009 s^−n^) reflects the fast kinetic evolution of the water contact angle.

The water uptake as a function of time exhibited increasing trends reaching plateau values after 40 and 120 h for our inorganic films obtained by lower (0.5 and 1.5 wt.%) and larger (3.3 and 5 wt.%) PT_Hal concentrations. This mismatch could be related to the different thickness and density structure of the films. More interestingly, it was detected that the water uptake at the saturation time is the smallest (ca. 30%) for thickest PT_Hal film with respect to the other films, which present water uptakes ranging between 40 and 50%. These results might be attributed to the compact fibrous network of PT_Hal films with the largest halloysite amount that can reduce the water absorption (all samples exhibit a mass change of at least 30%).

#### 2.1.2 Mechanical and Optical Properties

**Figure 4:**
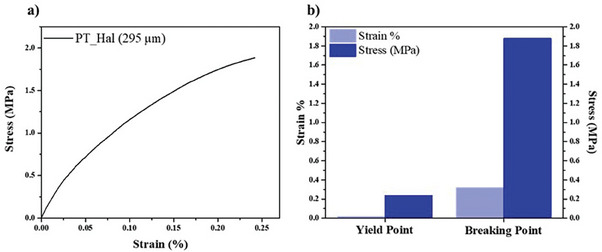
Mechanical properties of PT_Hal film prepared by the 5 wt.% aqueous dispersion. a) Stress versus strain curve obtained by tensile test. b) Stress and strain data at yield and breaking points.

The thickest PT_Hal film evidenced compactness and mechanical sustainability from a macroscopic viewpoint. These performances were quantitatively evaluated by performing tensile strength tests with Dynamic Mechanical Analysis (DMA) under a stress ramp mode. Films formed by PT_Hal amounts lower than 5 wt.% were fragile and therefore not tested. The obtained stress versus strain curve for PT_Hal 5 wt.% film and the calculated tensile parameters for yield and breaking points are displayed in **Figure** **4**.

As generally observed for thin films, the curve presented two regions that are related to elastic and plastic behaviors. The elastic part (linear stress vs strain trend) was observed up to strain and stress values of 0.01% and 0.2 MPa, which indicate the tensile properties at yield point (transition from elastic to plastic). The slope of the stress versus strain curve in the elastic region allowed us to calculate Young's modulus (ca. 1710 MPa), which is higher as compared to those detected for thin biopolymeric films.^[^ ^60^, ^61^ ^]^ The analysis of this curve provided also stress at the breaking point (1.88 MPa) and ultimate elongation (0.24%). Moreover, it was calculated the stored energy (650 kJ m^−3^) up to the film breaking from the integration of the area under the stress versus strain curve. Similar strength results were reported for lignocellulose paper sheets reinforced with halloysite nanotubes.^[^ ^62^ ^]^

**Figure 5:**
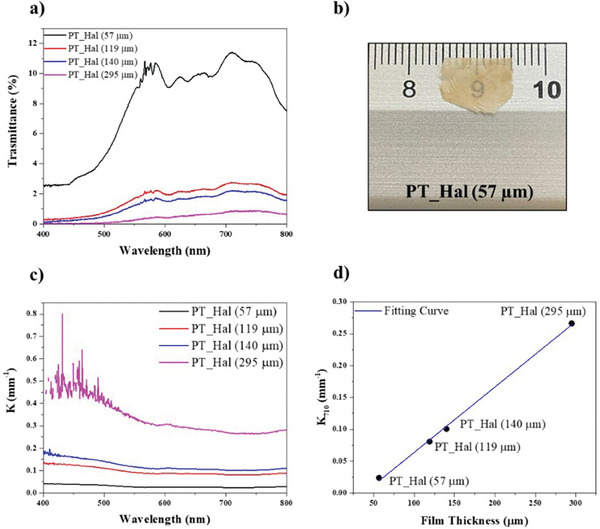
Optical properties of PT_Hal‐based films with variable thickness. a) Transmittance spectra of PT_Hal films. b) Optical image of the film with the smallest thickness (57 µm) c) Linear attenuation coefficient as a function of wavelength for PT_Hal films. d) Relation between the linear attenuation coefficient at 710 nm (K_710_) and the thickness for the PT_Hal films.

In addition, the optical characteristics of these inorganic films were investigated in order to evaluate their potential applications as filters in optoelectronic devices,^[^ ^63^ ^]^ anti‐reflective coatings,^[^ ^64^ ^]^ and energy‐efficient windows that reduce glare and heat gain.^[^ ^65^ ^]^ It was studied the transparency of PT_Hal‐based films prepared by dispersions with variable halloysite concentration by determining their transmittance within the visible region (**Figure** **5**a).

It was found that the transmittance is inverse proportional to the thickness of the PT_Hal film. Accordingly, Figure 5b shows that the inorganic film with the smallest thickness (57 µm) exhibits a moderate degree of transparency. It should be noted that the transmittance spectra show some peaks for wavelength ranging between 550 and 800 nm that could be related to Bragg reflections. Similar observations were published for inorganic films formed by nanocrystalline Zn_2_TiO_4_.^[^ ^66^ ^]^ The interference fringes were asymmetrically and arranged more densely in the region 550–600 nm (Figure 5a).

The linear attenuation coefficient (*K*) of the films was calculated according to Equation (2):

(2) $$K=A/\;\left(2.3\cdot D\right)$$

where *A* and *D* are the absorbance and the thickness of the films. Contrary to the transmittance (T), the *K* values are not mathematically dependent on the film thickness. Nevertheless, Figure 5c shows that the increase in the thickness shifted the K spectra to larger values. These results might be related to the variations of the structure of the films on the dependence of the PT_Hal concentration used during the casting procedure. In particular, the increase of the thickness could determine an enhancement of the density of long halloysite nanotubes forming the fibrous network. Interestingly, Figure 5d highlights a linear correlation between the attenuation coefficient at a fixed wavelength (710 nm) and the film thickness. This linear fitting could be used to control the light attenuation capacity of inorganic films by changing their thickness based on the casted PT_Hal dispersion concentration.

### 2.2 Applications of Inorganic Films Based on Long Patch Halloysite

**Figure 6:**
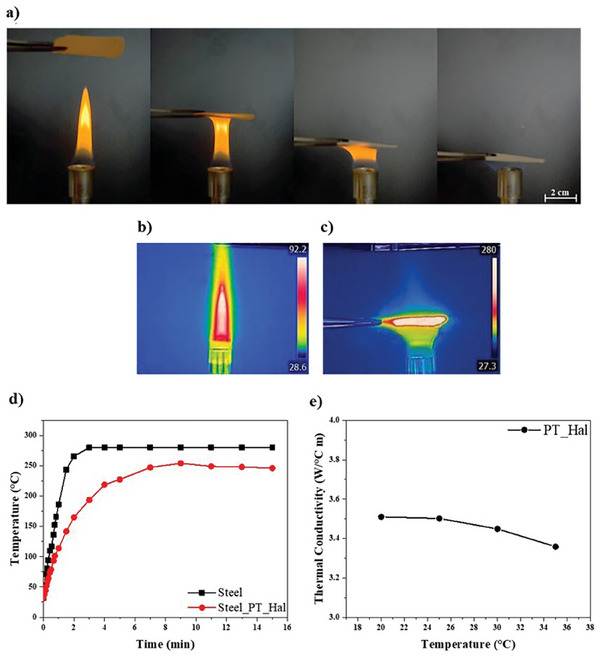
Fire resistance and heat barrier capacity of PT_Hal film with thickness equals to 295 µm. a) Flame resistance test on PT_Hal sample. b) Thermal image of flame. c) Thermal image of PT_Hal film (thickness of 295 µm) exposed to the flame for 30 min. d) Temperature versus time profile for the steel surface and a steel surface with PT_Hal film under identical thermal exposure to evaluate the protective effect of the film. e) Thermal conductivity of PT_Hal film (295 µm) in the temperature range between 20 and 35 °C.

Thin films based on inorganic components can be perspective because of their fire‐resistance capacity.^[^ ^67^, ^68^ ^]^ The development of hydrophilic and anti‐flammable films is a challenging task for industrial purposes as reported in recent works.^[^ ^69^, ^70^ ^]^ In this regard, PT_Hal films are promising being that the refractory nature of hydrophilic halloysite clay limits the heat conduction.^[^ ^71^ ^]^ It should be noted that the surface hydrophilicity of PT_Hal films was demonstrated by water contact angle data (Figure 3a). Our fire resistance tests evidenced that PT_Hal films are effective to block the flame propagation preventing any burning processes (**Figure** **6**a). Interestingly, the films did not evidence any macroscopic changes (deterioration and/or colorimetric alterations) after the flame tests. PT_Hal cast films preserved the robustness and stability through their flame exposure. One can state that these inorganic films have much better flame‐retardant properties than any organic‐containing composite films. The film's flame resistance is also confirmed by thermal images (Figure 6b,c). Interestingly, preservation of low temperature above the film which is exposed to the flame was detected. Specifically, the temperature at the distance of 1 cm from the PT_Hal film was measured for 30 min. As shown in Supplementary Information (Figure S2, Supporting Information), the temperature is nearly constant over the time within the range 40–45 °C. Moreover, the heat barrier capacity of the inorganic film was studied by using the approach sketched in Supplementary Information (Figure S3, Supporting Information). In particular, the kinetic evolution of the temperature of a steel specimen exposed to flame was determined. These experiments were performed on steel samples before and after the protective coating by the PT_Hal film. As a general result (Figure 6d), the temperature versus time trend showed an exponential increasing trend reaching a plateau after a few seconds from the flame exposure. It is worth noting that the heating rate of the steel is slowed down by the presence of PT_Hal film in agreement with the slope reduction of the temperature versus time trend. Accordingly, the constant temperature value was achieved after ca. 3 and 8 min for bare and protected steel specimens, respectively. Interestingly, the temperature at a plateau of the steel was reduced by ca. 40 °C (from ca. 280 to 240 °C) due to the coating protection with the inorganic film. It should be noted that the strongest protection effect was estimated after 3 min of flame exposure as evidenced by the highest temperature difference (87 °C, from 280 to 193 °C) between bare and coated steel. These results demonstrate that PT_Hal‐based film acts as an effective heat and fire barrier with potential applications in aerospace, where materials are often exposed to flames or high temperatures.

Additionally, the thermal conductivity of PT_Hal (Figure 6e) was determined following a method discussed by Marcus et al.^[^ ^72^ ^]^ based on Modulated Differential Scanning Calorimetry (MDSC) with direct measurements of the reversing heat capacities at 20, 25, 30, and 35 °C (Figure S3, Supporting Information). Across this temperature range, the conductivity remains relatively constant, with a value of ≈3.5 W·(°Cm^−1^). This result is in good agreement with the reported thermal conductivity data of nanoclays.^[^ ^73^, ^74^, ^75^ ^]^

In addition to their fire resistance, we suggest PT_Hal films as precursors of functional materials. The treatment procedures were carried out for the thickest PT_Hal film, which exhibited reliable mechanical resistance. The films' alkaline activation allowed us to fabricate geopolymers with even high thermo‐resistance and enhanced porosity, which can be exploited for CO_2_ storage. The produced geopolymer possesses an open and porous structure that favors CO_2_ capturing. Experimental data on the CO_2_ capture capacity of PT_Hal film before and after geopolymerization are presented in paragraph 2.4.

In the other approach, a high‐temperature 1050 °C treatment was used to convert the inorganic film into a ceramic, kind of similar to clay brick formation. The outcomes of these treatments offer insights into the films evolving with different modification processes, demonstrating how manipulating the materials structure can be leveraged to achieving specific objectives.

Moreover, both geopolymerization and thermal treatment were carried out on the PT_Hal film with the smallest thickness (Figure 5b) to evaluate the effects on the transparency.

### 2.3 Geopolymers and Ceramics Obtained by PT_Hal Film Treatments

#### 2.3.1 Effects of the Treatments on the Structural Characteristics of PT_Hal Film

**Figure 7:**
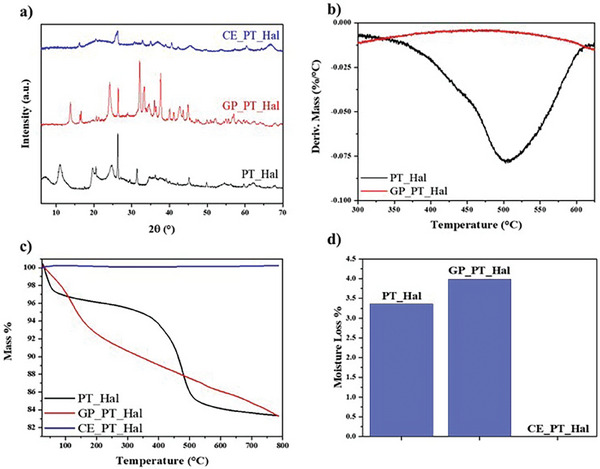
Effects of the treatments on the structural and thermal characteristics of PT_Hal films. a) XRD spectra of PT_Hal, GP_PT_Hal and CE_PT_Hal. b) Comparison of differential thermogravimetric curves for PT_Hal and GP_PT_Hal materials within the temperature range between 300 and 600 °C. c) Thermogravimetric curves of PT_Hal, GP_PT_Hal, and CE_PT_Hal within the whole investigated temperature interval. d) Moisture losses of PT_Hal, GP_PT_Hal and CE_PT_Hal materials.

The effects of the treatments on PT_Hal film were investigated by X‐ray diffraction (XRD), which allowed us to evaluate the formation of the geopolymer (GP_PT_Hal) and ceramic (CE_PT_Hal). As shown in **Figure** **7**a, the XRD curves of PT_Hal before treatment revealed the typical diffraction pattern of halloysite clay nanotubes.^[^ ^27^ ^]^

Specifically, we detected XRD peaks centred at 2θ equal to 11.02°, 19.68°, 24.80°, 29.54°, 34.84°, 39.14°, 54.60°, and 62.14° corresponding to (001), (100), (002), (110), (003), (210) and (300) planes, respectively.^[^ ^49^, ^74^ ^]^ The (001) plane corresponds to a basal spacing of 8 Å, which is slightly different with respect to the value (7 Å) reported for pristine halloysite.^[^ ^76^ ^]^ This phenomenon was explained by continuously collecting XRD patterns during in situ heating. It was observed that the dehydration process produces some irregular d‐spacings that are attributed to the interference of hydrated and dehydrated tubes.^[^ ^49^ ^]^ In addition, crystalline phases of quartz and aluminum oxide (XRD peaks at 2θ = 26.34° and 31.42°, respectively) were observed as residues from the purification process.

For the XRD spectrum of GP_PT_Hal (Figure 7a), it was observed a broad band between 20° and 40° arising from the amorphous structure in agreement with the halloysite geopolymerization.^[^ ^77^ ^]^ Accordingly, thermogravimetry confirmed the actual conversion of PT_Hal film to geopolymeric material. Contrary to PT_Hal, the differential thermogravimetric curve of GP_PT_Hal film (Figure 7b) did not show any signal between 350 and 550 °C indicating that the mass loss due to the dehydroxylation of aluminum inner sheets did not occur.^[^ ^60^ ^]^ This observation is a clear indication of the alkaline activation of PT_Hal and the formation of geopolymer as reported elsewhere.^[^ ^60^, ^78^ ^]^ In addition, the quantitative analysis of thermogravimetric curves (Figure 7c) allowed us to calculate the influence of the geopolymerization on the moisture loss of PT_Hal film by the determination of the mass losses between 25 and 120 °C that reflect the amounts of water molecules physically adsorbed onto the nanotubes surfaces. The obtained data (Figure 7d) evidenced a reduction of the hydrophilicity of GP_PT_Hal with respect to PT_Hal. According to the XRD spectrum (Figure 7a), GP_PT_Hal contains traces of silica, quartz, tridymite, and sodium oxide alongside the presence of some aluminosilicate crystalline phases, such as Al_3_Si_2_O_7_ (OH)_3_ and Na_6_(AlSiO_4_)_6_, which can be related to residues from the halloysite alkaline activation by NaOH.

Regarding the ceramic CE_PT_Hal sample without any alkaline treatment, the XRD spectrum (Figure 7a) exhibited three crystalline phases (quartz, mullite, and aluminum oxide) along with several broad bands between 15° and 50°, indicating the presence of an amorphous fraction in the material. As expected, ceramic material did not show any mass losses within the investigated temperature range because CE_PT_Hal was obtained by 1050 °C heating treatment.

**Figure 8:**
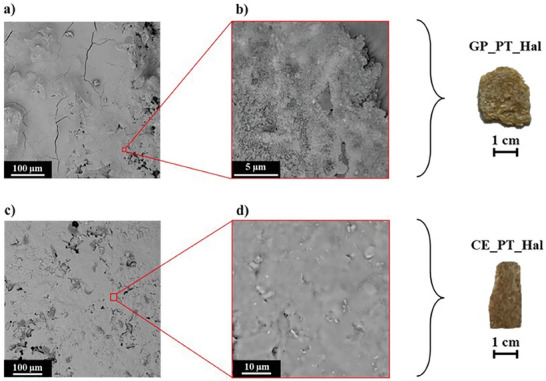
Morphology of geopolymer and ceramic obtained by the treatments of PT_Hal film. SEM images and optical photo of GP_PT_Hal a,b) CE_PT_Hal c,d) materials.

As shown at SEM images (**Figure** **8**), geopolymerization and heating treatment induced significant variations on the microscopic structure of the original PT_Hal film (Figure 2). Contrary to the initial nanoclay film, the fibrous morphology due to the arrangement of long patch nanotubes was not observed in both GP_PT_Hal and CE_PT_Hal materials. The geopolymeric material exhibited an inhomogeneous and porous morphology (Figure 8a,b). SEM images evidenced the presence of irregular nanometric particles, which are typical in bulk geopolymers obtained by the alkaline activation of commercial halloysite clay.^[^ ^60^ ^]^ The GP_PT_Hal sample presented pores with an average size of ca. 5 µm. On the other hand, it was observed that the ceramic CE_PT_Hal surface is uniform and compact without any porosities (Figure 8c,d). In addition to SEM micrographs, Figure 8 displays the macroscopic optical images of halloysite geopolymer and ceramic films.

#### 2.3.2 Hydrophilicity and Optical Properties of Halloysite Geopolymer and Ceramic

**Figure 9:**
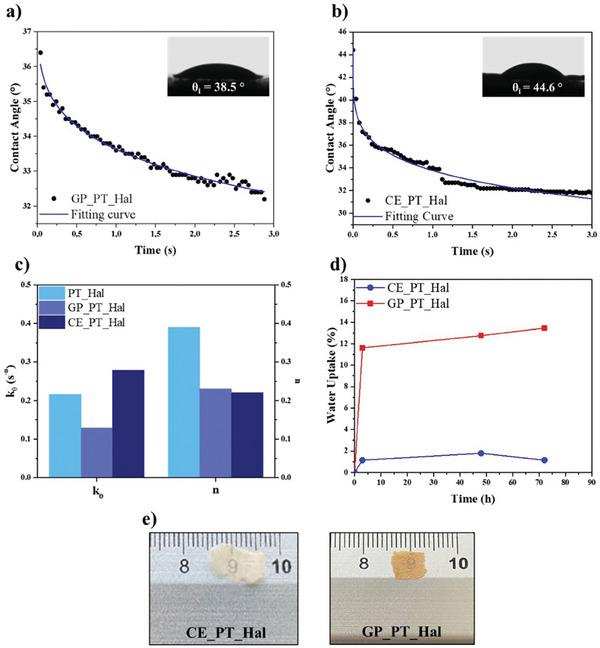
Hydrophilicity and optical properties of geopolymer and ceramic. a,b) Water contact angle as a function of time and images of the water droplets immediately after their deposition for GP_PT_Hal and CE_PT_Hal materials. c) Kinetic parameters obtained by the fitting of contact angle versus time trends through Equation (1) for PT_Hal, GP_PT_Hal, and CE_PT_Hal samples. The relative error for PT_Hal and CE_PT_Hal is ca. 5% while for GP_PT_Hal is ca. 10%. d) Water uptake as a function of time for GP_PT_Hal and CE_PT_Hal. e) Optical photos of geopolymer and ceramic obtained by the treatments of the PT_Hal film with the smallest thickness (57 µm).

The wettability and the water uptake capacity of both geopolymer and ceramic were studied to explore the influence of the treatments on the hydrophilic characteristics of PT_Hal film. As shown in **Figure** **9**a,b, the kinetic evolution of the water contact angle presents an exponential decay function for both GP_PT_Hal and CE_PT_Hal materials.

As reported for PT_Hal film (Figure 3a), the contact angle versus time trends were fitted using Equation (1) allowing us to determine the rate constant and the mechanism that affects the interactions between the water droplet and the materials surface. Figure 9c compares the fitting parameters as well as the initial contact angle values of geopolymer and ceramic with those for their precursor (PT_Hal film). GP_PT_Hal exhibited the fastest kinetic evolution of the water contact angle in agreement with the largest rate constant. As compared to PT_Hal film, a reduction of the exponential parameter n was estimated for both GP_PT_Hal and CE_PT_Hal samples.

Figure 9c evidences that both geopolymerization and thermal treatment induce the surface hydrophobization of the film. As reported in literature,^[^ ^61^ ^]^ the wettability characteristics are related to the chemical composition and roughness of the surfaces. Halloysite is a hydrophilic material because of the hydroxyl groups on its surface.^[^ ^79^ ^]^ The geopolymerization process of PT_Hal film caused a reduction in the hydroxyl groups creating the alumino‐silica network structure and consequently, the decrease of the surface hydrophilicity.^[^ ^80^ ^]^ This agrees with the water contact angle of GP_PT_Hal (38.5°), which is slightly lower than that of PT_Hal film (30.2°). On the other hand, the surface hydrophobization of CE_PT_Hal can be attributed to roughness enhancement as shown by SEM images (Figure 8c,d).

According to the contact angle data, the water uptake (Figure 9d) evidenced that both geopolymerization and thermal treatment generated a reduction of the hydrophilicity of PT_Hal film. In detail, it was calculated that the water uptakes at saturation point are ca. 12 and 2% for the geopolymeric and ceramic materials, respectively. These values are lower with respect to that (ca. 30%) of their precursor, which was the PT_Hal film with thickness of 295 µm. Moreover, it was observed that the kinetics of water absorption is significantly faster for GP_PT_Hal and CE_PT_Hal in comparison with the initial PT_Hal film. The water uptakes reached their maximum values after 2 h for both treated films, while the saturation point was achieved after 120 h for untreated PT_Hal film (Figure 3b).

To evaluate the macroscopical impact of the treatments, optical images of CE_PT_Hal and GP_PT_Hal materials are presented in Figure 9e. The high‐temperature process enabled the production of thin and ceramic demonstrating that the thermal treatment did not affect the ability of PT_Hal to transmit light. These suggest that the materials can maintain their optical properties even after being subjected to high temperatures. In contrast, precursor GP_PT_Hal appeared opaque highlighting that the geopolymerization altered the films transparency.

### 2.4 Halloysite Geopolymer Films Efficiency for CO_2_ Capture

**Figure 10:**
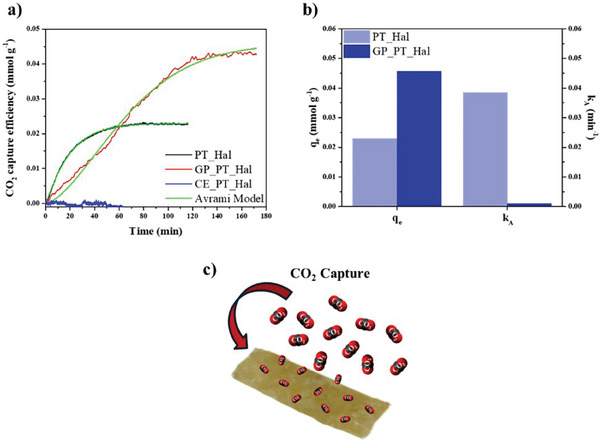
CO_2_ capture of PT_Hal‐based inorganic film, geopolymer, and ceramic. a) CO_2_ capture efficiency as a function of time for PT_Hal, GP_PT_Hal, and CE_PT_Hal. b) Adsorption parameters (q_e_, k_A_) obtained by the fitting analysis of CO_2_ capture efficiency versus time trends. The relative error is < 0.5%. c) Schematic representation for the CO_2_ captures onto the geopolymeric material.

Recently, the development of CO_2_ adsorbent materials based on biomass wastes^[^ ^81^, ^82^ ^]^ and natural inorganic materials^[^ ^83^, ^84^ ^]^ has gained increasing interest with the goal to achieve the net‐zero emissions by 2050, which is the main objective of the global CCUS projects proposed by International Energy Agency (IEA). In this regard, inorganic films and geopolymers obtained with this abundant natural nanoclay represent perspective eco‐friendly alternatives for CO_2_ capture from the air.^[^ ^85^, ^86^ ^]^ The CO_2_ sorption from PT_Hal and GP_PT_Hal clay materials was investigated. For comparison, the CO_2_ absorption experiments were conducted also by using ceramic films (CE_PT_Hal). The CO_2_ capture kinetics (**Figure** **10**) were determined by thermogravimetric measurements carried out in a CO_2_ saturated atmosphere.

According to its compact morphology shown by SEM images (Figure 8c,d), the ceramic material did not exhibit any sorption capacity toward CO_2_, while both PT_Hal and GP_PT_Hal were efficient as sorbents for CO_2_ capture. As displayed in Figure 10a, the kinetic trends of CO_2_ adsorption were successfully analyzed through the Avrami kinetic model, which is extensively used for the fitting analysis of CO_2_ adsorption onto solid materials, such as porous geopolymer,^[^ ^87^ ^]^ lithium silicates from demolition wastes^[^ ^88^ ^]^ and metal–organic framework.^[^ ^89^ ^]^ Based on the Avrami kinetic model, the kinetic evolution of the CO_2_ adsorption (*q*_t_) can be described by the following Equation:

(3) $$q_{t}=q_{e}\left(1-e^{\left(k_{A}t\right)n_{A}}\right)$$

where *q*_t_ is the CO_2_ adsorption value at equilibrium, whereas *k*_A_ and *n*_A_ are the Avrami rate constant and the Avrami parameter, which is related to the induction time of the investigated process.^[^ ^89^, ^90^ ^]^ The calculated *q*_e_ and *k*_A_ parameters are collected in Figure 10b. It was estimated that *q*_e_ of GP_PT_Hal is ca. two times larger with respect to the inorganic film in agreement with the enhancement of the porosity evidenced by SEM images (Figure 8a,b). The CO_2_ capture process of GP_PT_Hal is significantly slower than that of PT_Hal as highlighted by the *k*_A_ values. Additionally, it was calculated that the Avrami exponent n is ca. 1 for PT_Hal film indicating that the CO_2_ adsorption might be described also by a pseudo‐first order model,^[^ ^91^ ^]^ which provided similar results in comparison with the Avrami model. Specifically, the values of kinetic constant (*k*_1_) and *q*_e_ are equal to 0.04890 ± 1.5 × 10^−5^ min^−1^ and 0.02318 ± 1.7 × 10^−6 ^mmol g^−1^, respectively. Oppositely to PT_Hal, the Avrami exponential parameter for GP_PT_Hal is significantly larger than 1 evidencing that an induction time is required before the CO_2_ capture on geopolymer takes place. Specifically, it was calculated an Avrami exponential parameter of 1.580 ± 0.01 for GP_PT_Hal. This finding might indicate that the CO_2_ capture onto the geopolymeric material is simultaneously driven by both chemisorption and physisorption mechanisms as reported elsewhere.^[^ ^92^ ^]^ According to the Avrami fitting parameters, we can conclude that the geopolymerization process enhances the CO_2_ capture capacity of the inorganic film due to the increase of the pore volume as sketched in Figure 10c. Moreover, the CO_2_ absorption rate was reduced, and the capture mechanism was altered.

## 3 Conclusion

This work represents the first approach to fabricate inorganic films by self‐assembling of very long halloysite clay nanotubes (Hal) using the aqueous casting procedure. To this purpose, purified patch halloysite (PT_Hal) was employed because of its peculiar geometrical characteristics, including larger length and much higher aspect ratio thus contrasting with commercial clay nanotubes. The interconnection and tangling of Patch halloysite nanotubes determined the formation of self‐standing clay films with a fibrous morphology. The properties of these halloysite films are related to their thickness, which can be easily controlled by changing the PT_Hal concentration of the aqueous dispersion used in the casting protocol. The film with the largest thickness (295 µm) exhibited the lowest water uptake capacity and the highest light attenuation coefficient and tensile performances, which are comparable with respect to polymeric materials in terms of elastic modulus (1710 MPa) and stored energy up to break (650 kJ m^−3^). Due to their inorganic composition, PT_Hal films were efficient as fire‐resistant materials preventing flame propagation.

Further, these inorganic clay films were converted to advanced materials with specific functionalities by thermal treatment or alkaline activation, which allowed us to fabricate ceramic (CE_PT_Hal) and high porous and thermo‐stable geopolymer (GP_PT_Hal), respectively. Both treatments induced a significant hydrophobization of the initial PT_Hal film that can be related to roughness enhancement and variations of the surface chemistry. Due to the high porosity, the geopolymeric material was effective as a CO_2_ sorbent. The absorption capacity of GP_PT_Hal was twice as large as compared to the corresponding PT_Hal film. Produced geopolymeric material is a perspective for CO_2_ capture and storage reducing atmospheric pollution.

## 4 Experimental Section

### Purification of Patch Halloysite Nanotubes

Raw Patch Halloysite is a kind donation provided by Dr. Keith Norrish from his collection and research on Patch Halloysite (CSIRO Soils, Adelaide). The PT_Hal sample was collected at 8 m depth, under a thick lateritic cap, and composed of long laths.^[^ ^93^ ^]^ This specimen has a very pale white–blue translucent appearance, high moisture contents, and presents an atypical structure in that the partially collapsed tubes are up to 30 µm in length, with uniform diameter and very thin walls.

The purification of raw Patch Halloysite was carried out following a method described elsewhere.^[^ ^26^, ^49^ ^]^ First, the sample was ground using a mortar to reduce the grain size and then dispersed in distilled water. The pH was adjusted using a 0.1 m NaOH solution to achieve values between 7.5 and 8, obtaining a clay dispersion. The impurities were separated through sedimentation and several cycles of filtration. The dispersion was then dried at 80 °C for 24 h to gain the purified Patch halloysite.

### Preparation of PT‐Hal‐Based Films

**Figure 11:**
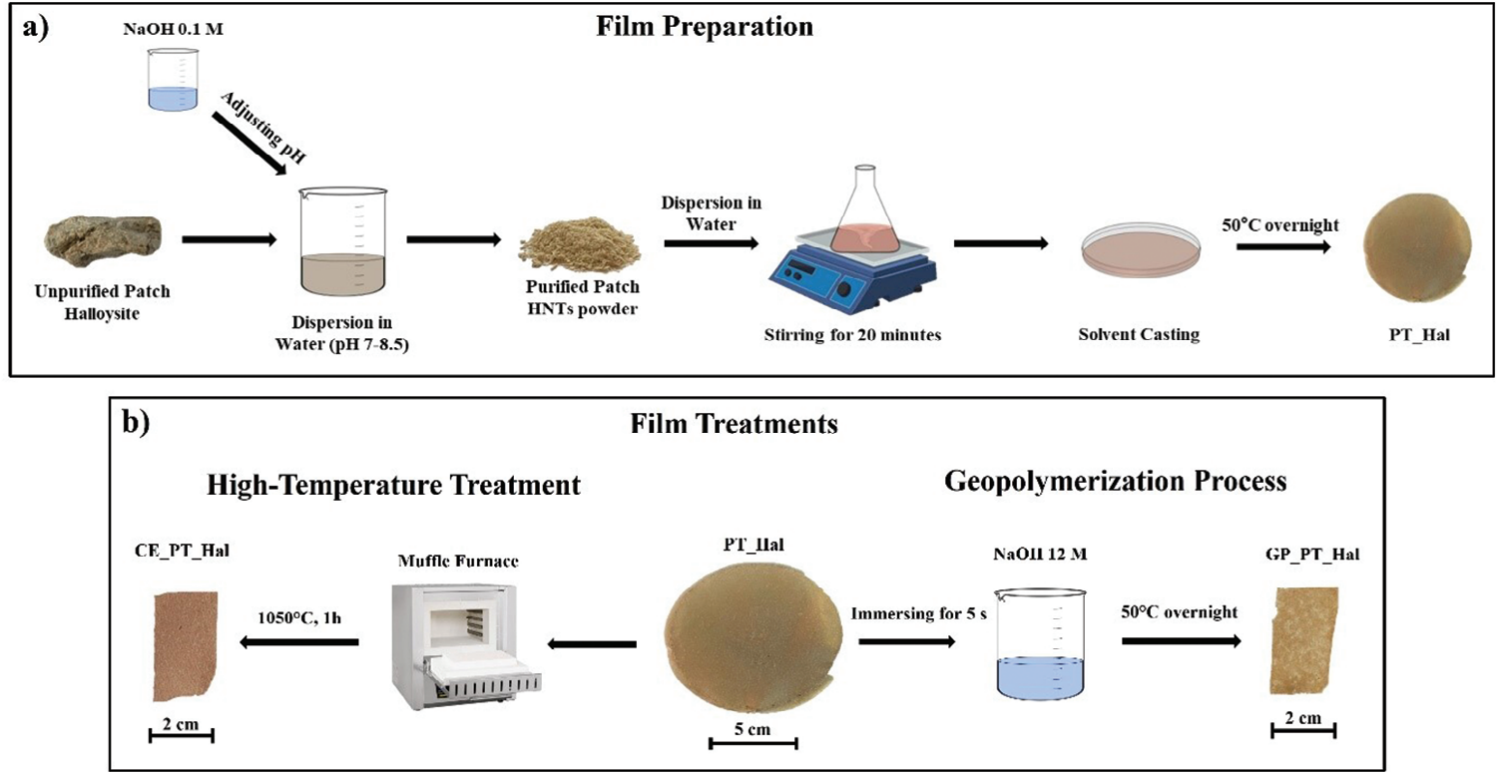
Preparation of inorganic film based on patch halloysite nanotubes and their consequent conversion to geopolymer and ceramic. a) Schematic illustration of the preparation of PT_Hal‐based film. b) Sketch of the PT_Hal film treatments for the fabrication of geopolymer (GP_PT_Hal) and ceramic (CE_PT_Hal).

Inorganic films based on purified PT_Hal were prepared by the aqueous casting procedure (**Figure** **11**).

Initially, the PT‐Hal powder was dispersed in water at different concentrations (0.5, 1.5, 3.3, and 5 wt.%). The dispersions were magnetically stirred for 20 min, poured into a plastic dish (LDPE), and placed in an oven at 50 °C overnight to ensure complete water evaporation. Finally, the inorganic films were easily removed from the plastic support and stored in a desiccator at 25 °C and relative humidity of 75%.

The same casting procedure was tested by preparing aqueous dispersions of commercial halloysite provided by Sigma–Aldrich (S_Hal). As shown in Supporting Information (Figure S1, Supporting Information), s_Hal does not generate the formation of inorganic films due to their different geometrical characteristics with respect to PT_Hal. The two types of halloysite differ primarily in the length of the nanotubes, which usually depends on the specific geological deposit. Typically, s_Hal (obtained from Dragon Mine source) possesses a length of ca. 1 µm, with an external diameter of ca. 20–150 nm and an inner diameter of ca. 5–30 nm.^[^ ^26^ ^]^ SEM images of s_Hal are presented in Supporting Information. On the other hand, Patch Halloysite nanotubes are thinner and longer being that the PT_Hal length can reach up to 30 µm. According to these considerations, the formation of the film is likely due to the ability of Patch halloysite to arrange into fibrous and bird nest structures, which can support the film.^[^ ^27^ ^]^

### Conversion of PT_Hal Film to Geopolymer and Ceramic

The PT_Hal film prepared from the 5 wt.% aqueous dispersion was treated in two different ways to obtain a PT_Hal‐based geopolymer (GP_PT_Hal) and a ceramic material (CE_PT_Hal). As sketched in Figure 1b, GP_PT_Hal sample was fabricated by immersing the PT_Hal film within 12 m NaOH solution, which acts as alkaline activation of halloysite as reported elsewhere.^[^ ^60^ ^]^ The immersion time (10 s) was reduced compared to conventional geopolymerization protocols, which usually indicate 24 h,^[^ ^60^, ^94^ ^]^ in order to avoid the disintegration of the film structure. To this purpose, the NaOH/Hal ratio was larger than 1. Then, the sample was kept at 50 °C overnight for a complete water evaporation. As concerns the preparation of ceramic, the PT_Hal film was thermally at 1050 °C for 1 h using a muffle furnace.

The geopolimerization and thermal treatments were conducted also on the PT_Hal‐based film with the smallest thickness (57 µm) to evaluate the effects on the transparency.

### Materials Characterization

The XRD patterns were obtained from an X‐ray diffractometer (Rigaku, MiniFlex) with a CuKa radiation source including a nickel filter, and working at 40 kV and 15 mA. The wavelength of the X‐ray beam was 1.5406 Å, and the layer spacing of the samples was calculated by Bragg's equation, which can be expressed by the following Equation:

(4) nλ=2dsinθ

where *θ* is the angle that the outgoing beam forms with the crystalline layer, *λ* is the wavelength of the radiation, *d* is the distance between two adjacent layers and n can be 1,2 or 3. The angle for scanning ranged from 6° to 70° with a rate of 20° min^−1^ and a step of 0.02°.

Thermogravimetric experiments were carried out using a TGA550 apparatus (TA Instruments) under an inert atmosphere using nitrogen flows of 60 and 40 cm^3^ min^−1^ for the sample and the balance, respectively. Samples were heated in a platinum pan from room temperature to 800 °C with a scanning rate of 20 °C min^−1^.

Thermal images were collected by a FLIR E6‐XT infrared camera (FLIR Commercial Systems Inc.) with 240 × 180 pixels.

Morphological studies of purified PT_Hal sample were performed by ultra‐high resolution field emission scanning electron microscope (FE‐SEM, Hitachi SU8010). A thin layer of platinum was applied to coat the samples to avoid electrostatic charging. Further micrographs of Patch halloysite nanotubes were obtained using the transmission mode (STEM) of the same FE‐SEM instrument with a voltage of 30 kV.

Morphological investigations of inorganic films, geopolymers, and ceramics were conducted using a scanning electron microscope (SEM) (Desktop SEM Phenom PRO X PHENOM) with a magnification range of 160x to 350,000x and a voltage range between 4.8 and 20.5 kV. For the SEM analyses, each sample was preliminarily coated with a 3 nm layer of gold to prevent charging effects under the electron beam.

Water contact angle measurements were carried out using an optical contact angle apparatus (OCA 20, Data Physics Instruments, Filderstadt, Germany) implemented with a video measuring system, a high‐resolution CCD camera, and a high‐performance digitizing adapter. Data acquisition was conducted by SCA 20 software (Data Physics Instruments, Filderstadt, Germany). The water contact angle was measured using the sessile drop method. A water droplet of 10 ± 0.5 µL was gently placed on the film surface by a syringe pointed vertically downward onto the sample. The tests were conducted at a temperature of 25.0 ± 0.1 °C. Images were collected at a rate of 25 frames per second starting from the deposition of drop for 60 s.

Water uptake measurements were conducted using a desiccator containing a beaker filled with water to create an atmosphere with ≈99% humidity. The samples were placed inside and weighed at various time intervals. The water uptake values were calculated using Equation (5):

(5) $$\mathrm{Water}\;\mathrm{Uptake}\%=\left(\left(w_{i}-w_{t}\right)/w_{i}\right)\cdot 100$$

where *w*_i_ is the initial weight and *w*_t_ is the weight at time *t*.

A DMA Q800 (TA Instruments) was employed to investigate the tensile properties of the inorganic films. Specifically, the films were cut into rectangular shape and a ramp stress of 1 MPa min^−1^ at 25 ± 0.5 °C was performed.

Optical properties were investigated by transmittance experiments carried out at 25.0 ± 0.1 °C using a UV–vis spectrophotometer (Specord S600 Analytik Jena).

Thermal conductivity of PT_Hal material was evaluated using Modulated Differential Scanning Calorimetry (MDSC) by a DSC 25 (TA Instruments, Discovery Series) apparatus. The calibration was conducted using a sapphire standard. The experiments were conducted based on a quasi‐isothermal MDSC method with a temperature modulation of ± 1.00 °C and a cycle period of 120 s. Samples were equilibrated at 10 °C, followed by a 5 min isothermal period before data collection. Temperature was then increased in 5 °C steps, and the process was repeated over multiple cycles. Thermal conductivity (K) was calculated from the experimental data using the Equation (6):

(6) $$K=\left(2\pi C^{2}\right)/\left(C_{p}\cdot \rho \cdot A_{\mathrm{cs}}^{2}\cdot P\right)$$

where *C* is the apparent heat capacity, *C*_p_ is the specific heat capacity, *ρ* is the density of the sample, *A*_cs_ is the cross‐sectional area of the sample, *P* is the modulation period.

### CO_2_ Capture Tests

The CO_2_ capture capacity of PT_Hal, GP_PT_Hal, and CE_PT_Hal materials was investigated by using the TGA equipment (TGA 550 Discovery Series – TA Instruments). Initially, the adsorbent materials were equilibrated at 100 °C in a nitrogen‐inert atmosphere to remove moisture until a stable weight was achieved. Subsequently, the temperature was lowered to 35 °C, followed by the introduction of CO_2_ gas. Flow rates for both gases (nitrogen and CO_2_) were fixed at 60 and 40 cm^3^ min^−1^ for the sample and the balance, respectively.

## Conflict of Interest

The authors declare no conflict of interest.

## Supporting information



Supporting Information

## Data Availability

The data that support the findings of this study are available from the corresponding author upon reasonable request.
