# Supplementary material for: Flame‐Resistant Inorganic Films by Self‐Assembly of Clay Nanotubes and their Conversion to Geopolymer for CO2 Capture
Source: Small. 2024 Oct 7;20(51):2406812. doi: 10.1002/smll.202406812 (PMC11656676; doi:10.1002/smll.202406812)
Supplement: Supplementary file 1 — Supporting Information [file SMLL-20-2406812-s001.docx]

**Supplementary Information**

**Flame-Resistant Inorganic Films by Self-Assembly of Clay Nanotubes and their Conversion to Geopolymer for CO_2_ Capture**

Alessandro Lo Bianco,^a^ Martina Maria Calvino,^a^ Giuseppe Cavallaro,* Lorenzo Lisuzzo, Pooria Pasbakhsh, Stefana Milioto, Giuseppe Lazzara, Yuri Lvov


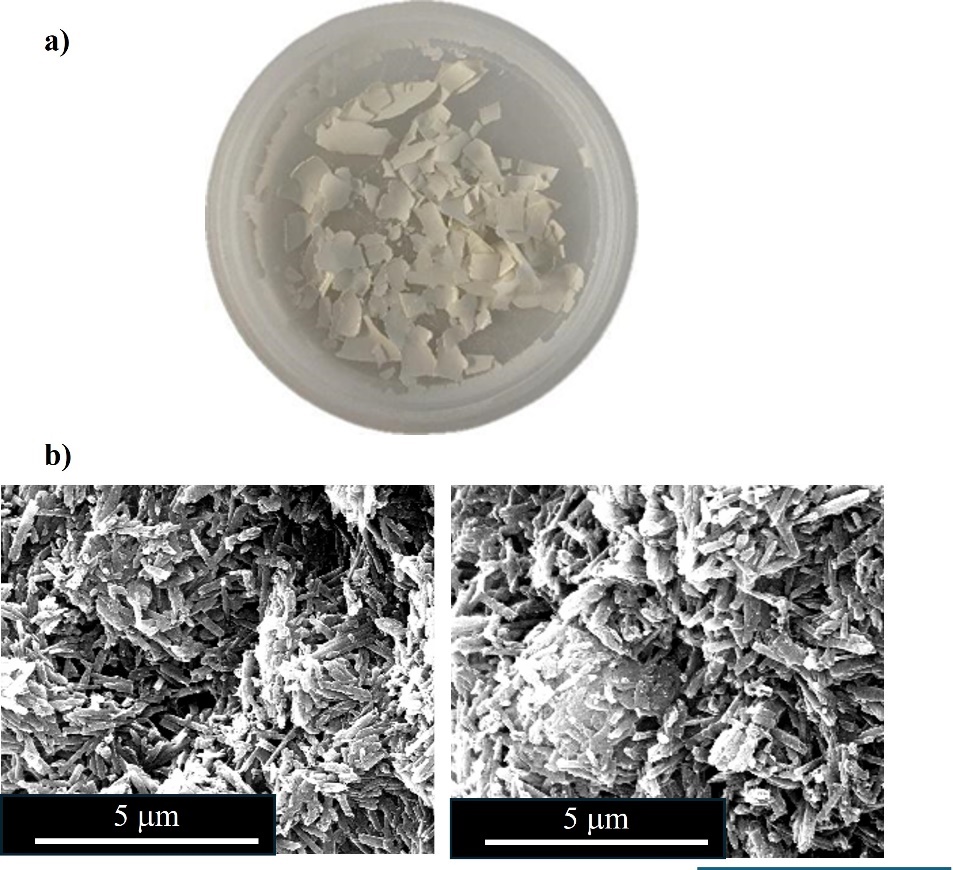


Figure S1. (a) Optical photograph of commercial halloysite from Sigma-Aldrich (S_Hal) after aqueous casting. (b) SEM images of S-Hal sample.

**Figure S2.** Temperature measured 1 cm above the PT_Hal film as a function of time.

**
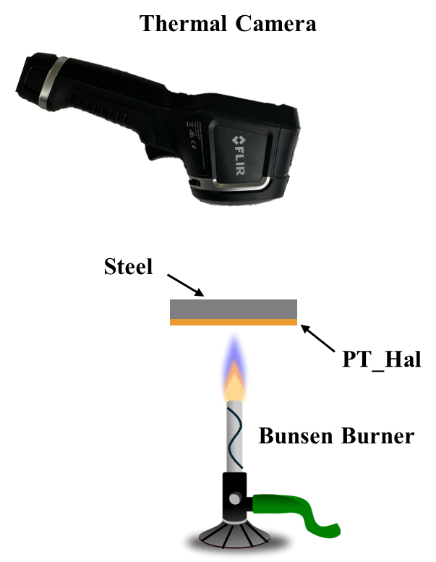
**

**Figure S3.** Illustration of the experimental setup to measure the temperature on the Steel surface

**
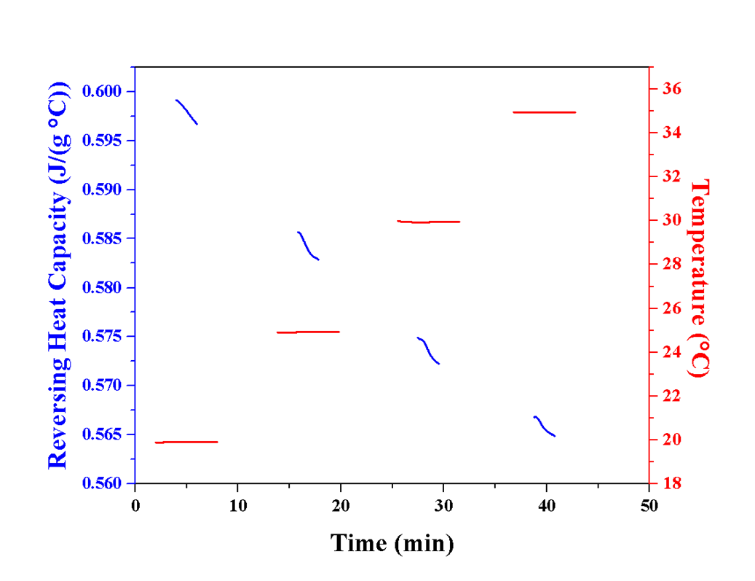
**

**Figure S4.** Reversing heat capacity plots obtained from MDSC experiments.
